# Supplementary material for: Pyruvate Dehydrogenase Kinase 1 inhibition mediated oxidative phosphorylation enhancement in cartilage promotes osteoarthritis progression
Source: BMC Musculoskelet Disord. 2023 Jul 20;24:597. doi: 10.1186/s12891-023-06585-6 (PMC10357736; doi:10.1186/s12891-023-06585-6)
Supplement: Supplementary file 1 — Additional file 1. [file 12891_2023_6585_MOESM1_ESM.zip › BP.Gsea.1653623667859/GOBP_INTRA_GOLGI_VESICLE_MEDIATED_TRANSPORT.html]

Details for gene set GOBP\_INTRA\_GOLGI\_VESICLE\_MEDIATED\_TRANSPORT[GSEA]

|  || Dataset | 55457\_collapsed\_to\_symbols.cls.cls #OA\_versus\_normal.cls.cls #OA\_versus\_normal\_repos |
| Phenotype | cls.cls#OA\_versus\_normal\_repos |
| Upregulated in class | OA |
| GeneSet | GOBP\_INTRA\_GOLGI\_VESICLE\_MEDIATED\_TRANSPORT |
| Enrichment Score (ES) | 0.61831415 |
| Normalized Enrichment Score (NES) | 2.155743 |
| Nominal p-value | 0.0 |
| FDR q-value | 0.004096886 |
| FWER p-Value | 0.059 |
Table: GSEA Results Summary

  

Fig 1: Enrichment plot: GOBP\_INTRA\_GOLGI\_VESICLE\_MEDIATED\_TRANSPORT      
 Profile of the Running ES Score & Positions of GeneSet Members on the Rank Ordered List

  

| SYMBOL | TITLE | RANK IN GENE LIST | RANK METRIC SCORE | RUNNING ES | CORE ENRICHMENT || 1 | COPE | COPI coat complex subunit epsilon [Source:HGNC Symbol;Acc:HGNC:2234] | 15 | 1.136 | 0.0995 | Yes |
| 2 | COPZ2 | COPI coat complex subunit zeta 2 [Source:HGNC Symbol;Acc:HGNC:19356] | 19 | 1.109 | 0.1976 | Yes |
| 3 | COG5 | component of oligomeric golgi complex 5 [Source:HGNC Symbol;Acc:HGNC:14857] | 38 | 1.012 | 0.2860 | Yes |
| 4 | VTI1B | vesicle transport through interaction with t-SNAREs 1B [Source:HGNC Symbol;Acc:HGNC:17793] | 336 | 0.732 | 0.3278 | Yes |
| 5 | COPZ1 | COPI coat complex subunit zeta 1 [Source:HGNC Symbol;Acc:HGNC:2243] | 421 | 0.697 | 0.3831 | Yes |
| 6 | COPB2 | COPI coat complex subunit beta 2 [Source:HGNC Symbol;Acc:HGNC:2232] | 504 | 0.658 | 0.4351 | Yes |
| 7 | GABARAPL2 | GABA type A receptor associated protein like 2 [Source:HGNC Symbol;Acc:HGNC:13291] | 776 | 0.575 | 0.4650 | Yes |
| 8 | NSF | "N-ethylmaleimide sensitive factor, vesicle fusing ATPase [Source:HGNC Symbol;Acc:HGNC:8016]" | 908 | 0.540 | 0.5028 | Yes |
| 9 | COG4 | component of oligomeric golgi complex 4 [Source:HGNC Symbol;Acc:HGNC:18620] | 966 | 0.525 | 0.5449 | Yes |
| 10 | GOLGA5 | golgin A5 [Source:HGNC Symbol;Acc:HGNC:4428] | 1016 | 0.514 | 0.5867 | Yes |
| 11 | RAB6A | "RAB6A, member RAS oncogene family [Source:HGNC Symbol;Acc:HGNC:9786]" | 1599 | 0.413 | 0.5782 | Yes |
| 12 | COG7 | component of oligomeric golgi complex 7 [Source:HGNC Symbol;Acc:HGNC:18622] | 1691 | 0.400 | 0.6066 | Yes |
| 13 | COPA | COPI coat complex subunit alpha [Source:HGNC Symbol;Acc:HGNC:2230] | 1959 | 0.366 | 0.6183 | Yes |
| 14 | TRAPPC3 | trafficking protein particle complex subunit 3 [Source:HGNC Symbol;Acc:HGNC:19942] | 2527 | 0.300 | 0.6009 | No |
| 15 | NAPA | NSF attachment protein alpha [Source:HGNC Symbol;Acc:HGNC:7641] | 2742 | 0.276 | 0.6087 | No |
| 16 | RAB33B | "RAB33B, member RAS oncogene family [Source:HGNC Symbol;Acc:HGNC:16075]" | 3673 | 0.188 | 0.5531 | No |
| 17 | COG2 | component of oligomeric golgi complex 2 [Source:HGNC Symbol;Acc:HGNC:6546] | 3767 | 0.180 | 0.5619 | No |
| 18 | GOSR2 | golgi SNAP receptor complex member 2 [Source:HGNC Symbol;Acc:HGNC:4431] | 5037 | 0.070 | 0.4695 | No |
| 19 | GOSR1 | golgi SNAP receptor complex member 1 [Source:HGNC Symbol;Acc:HGNC:4430] | 6166 | -0.018 | 0.3835 | No |
| 20 | COPG1 | COPI coat complex subunit gamma 1 [Source:HGNC Symbol;Acc:HGNC:2236] | 8289 | -0.178 | 0.2345 | No |
| 21 | RAB6B | "RAB6B, member RAS oncogene family [Source:HGNC Symbol;Acc:HGNC:14902]" | 8309 | -0.179 | 0.2488 | No |
| 22 | COPB1 | COPI coat complex subunit beta 1 [Source:HGNC Symbol;Acc:HGNC:2231] | 9447 | -0.269 | 0.1844 | No |
| 23 | NAPG | NSF attachment protein gamma [Source:HGNC Symbol;Acc:HGNC:7642] | 10671 | -0.390 | 0.1240 | No |
| 24 | TRAPPC10 | trafficking protein particle complex subunit 10 [Source:HGNC Symbol;Acc:HGNC:11868] | 11818 | -0.553 | 0.0840 | No |
Table: GSEA details [plain text format]

  

Fig 2: GOBP\_INTRA\_GOLGI\_VESICLE\_MEDIATED\_TRANSPORT      
 Blue-Pink O' Gram in the Space of the Analyzed GeneSet

  

Fig 3: GOBP\_INTRA\_GOLGI\_VESICLE\_MEDIATED\_TRANSPORT: Random ES distribution      
 Gene set null distribution of ES for **GOBP\_INTRA\_GOLGI\_VESICLE\_MEDIATED\_TRANSPORT**
